# Supplementary material for: The Performance of GALAD Score for Diagnosing Hepatocellular Carcinoma in Patients with Chronic Liver Diseases: A Systematic Review and Meta-Analysis
Source: J Clin Med. 2023 Jan 26;12(3):949. doi: 10.3390/jcm12030949 (PMC9918029; doi:10.3390/jcm12030949)
Supplement: Supplementary file 1 [file jcm-12-00949-s001.zip › jcm-2159271-supplementary.pdf]

Supplementary Materials

The Performance of GALAD Score for Diagnosing Hepatocellular Carcinoma in Patients with Chronic Liver Diseases: A Systematic Review and Meta-Analysis

A

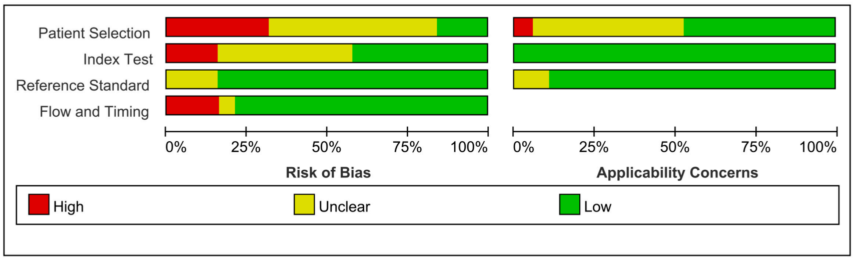

B

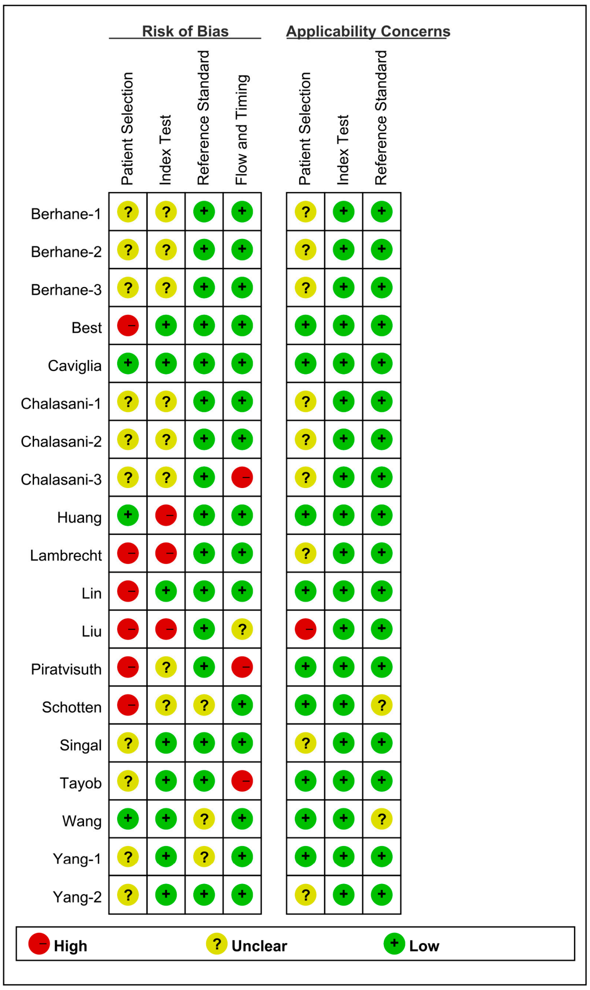

Figure S1. Quality evaluation of included studies. A) Methodological quality graph; B) Methodological quality summary.

A

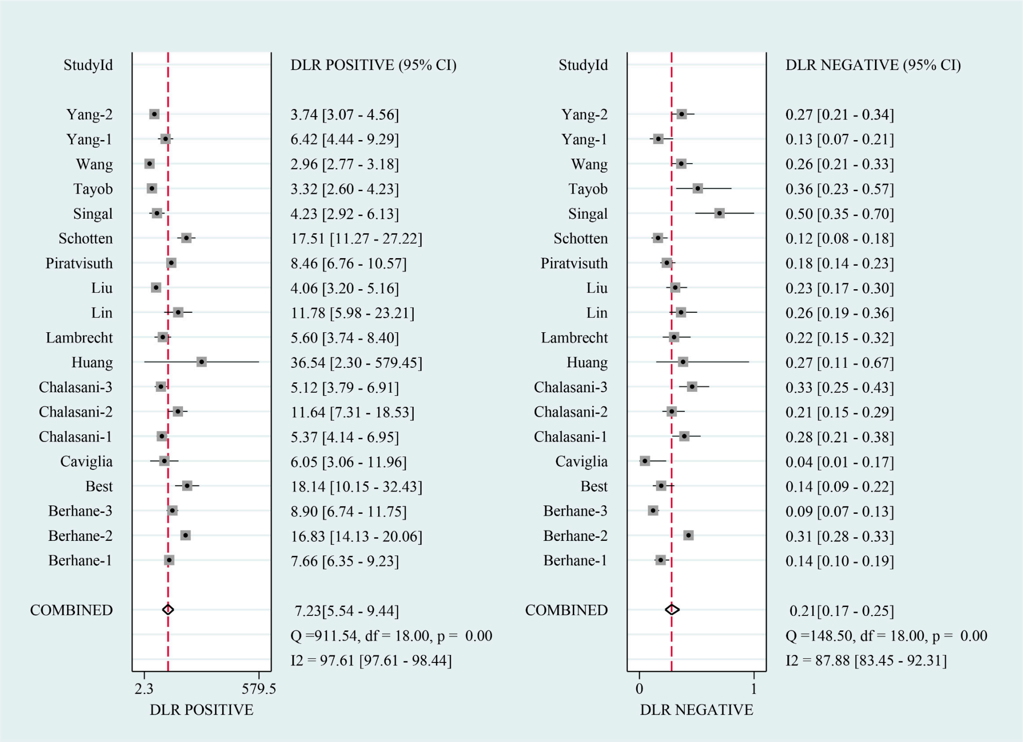

B

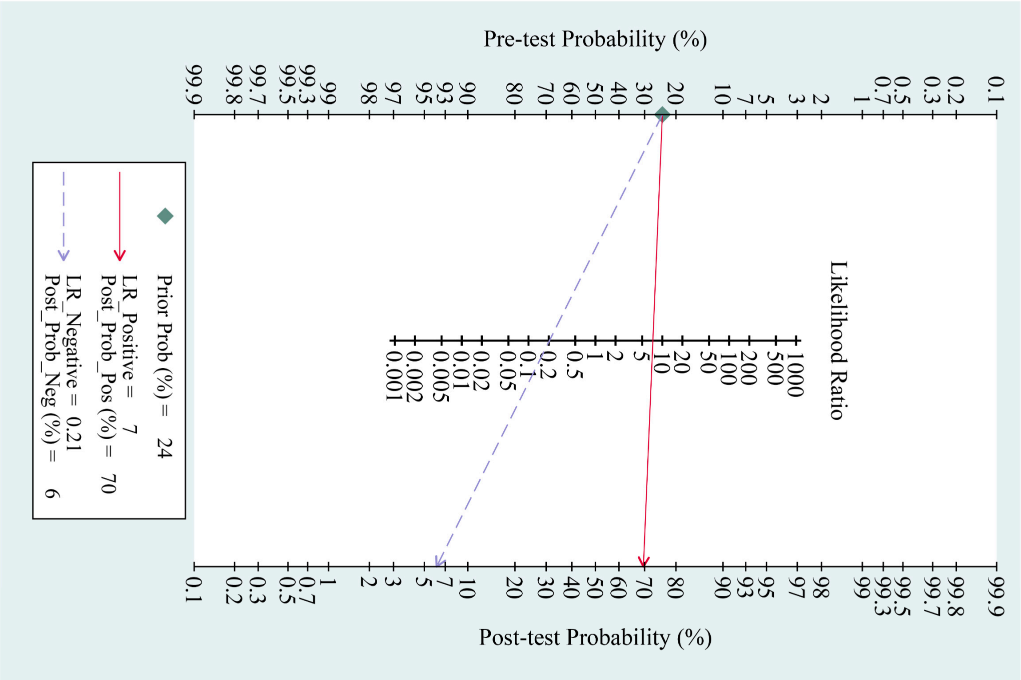

Figure S2. The pooled positive and negative likelihood ratio with Forest plots (A) and Fagan diagram (B) assessing the overall diagnostic value of GALAD score for discriminating any-stage hepatocellular carcinoma in chronic liver disease.

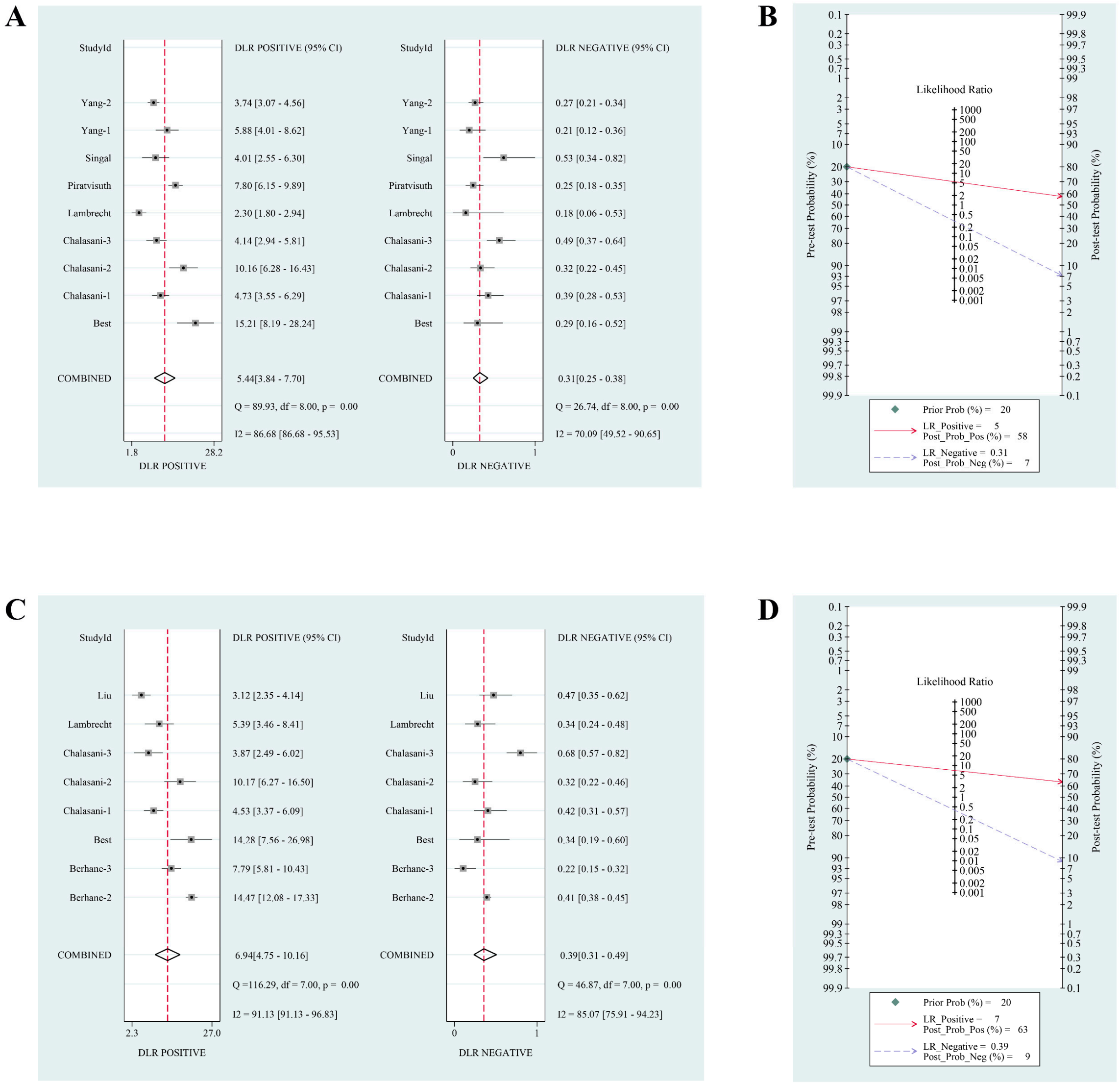

Figure S3. The pooled positive and negative likelihood ratio with Forest plots and Fagan diagram assessing the overall diagnostic value of GALAD score for discriminating early-stage hepatocellular carcinoma within Barcelona Clinic Liver Cancer 0/A staging (A, B) or within Milan criteria (C, D).

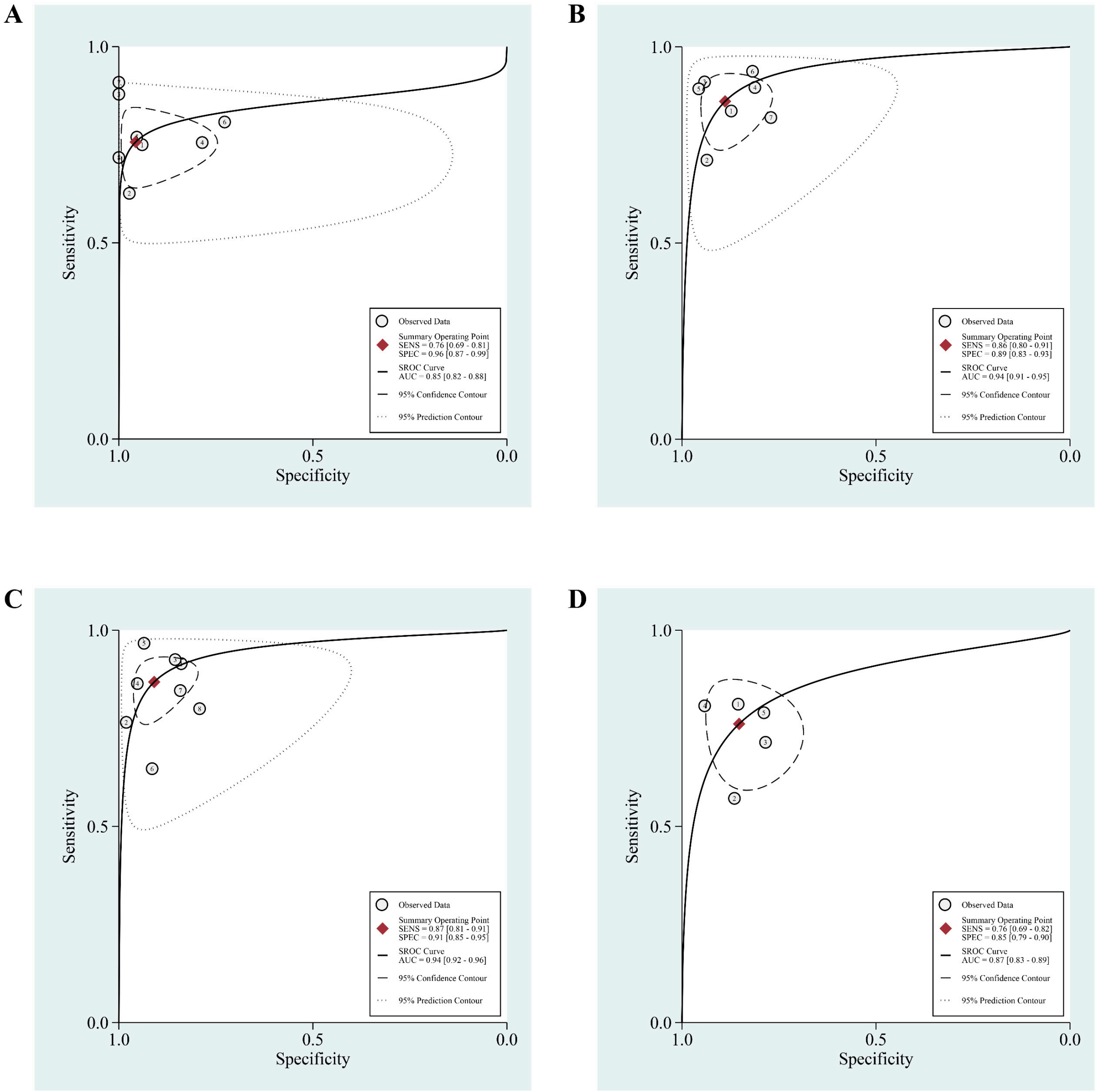

Figure S4. The summary receiver operating characteristic curves of GALAD score for discriminating hepatocellular carcinoma in patients with different etiologies. A) hepatitis B virus; B) hepatitis C virus; C) non-viral liver diseases; D) cirrhosis.

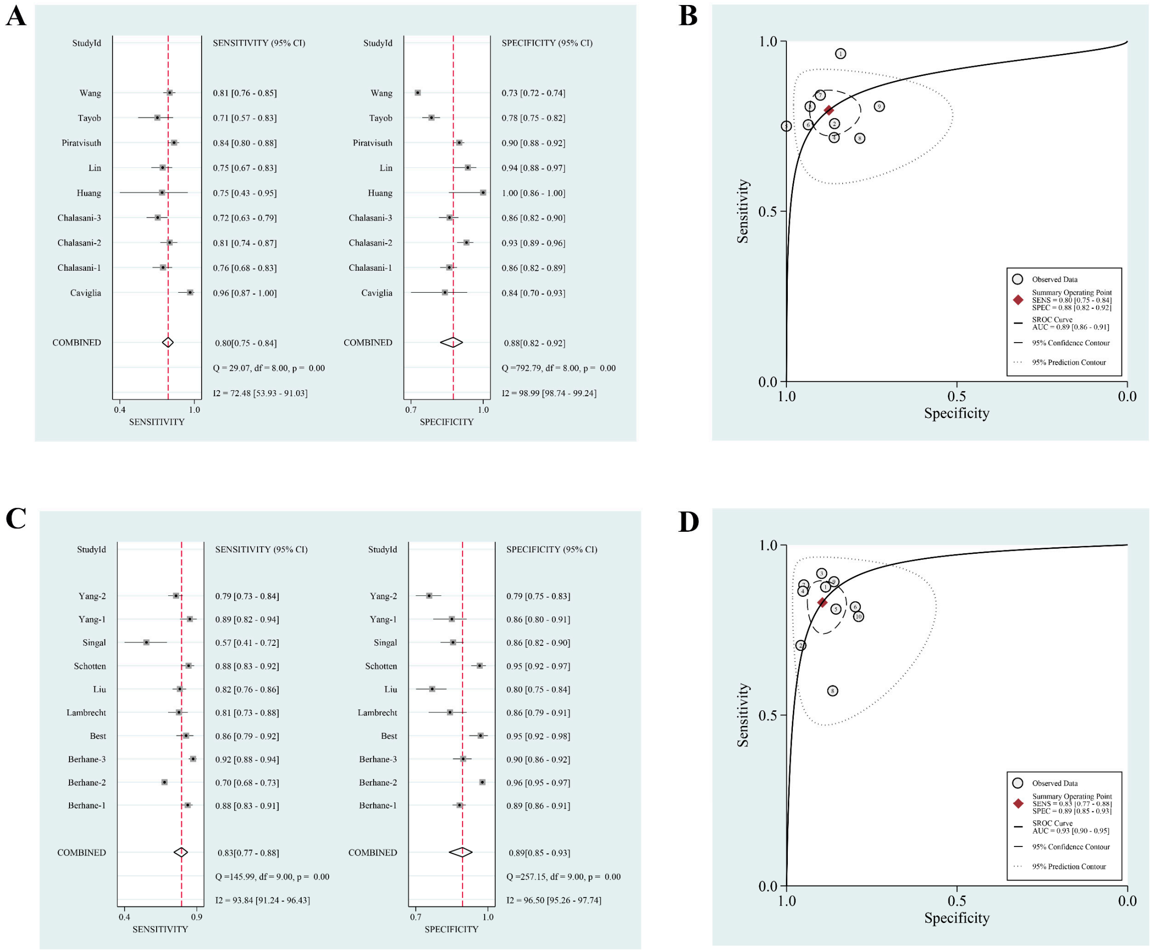

Figure S5. The pooled sensitivity and specificity with Forest plots and summary receiver operating characteristic curve of GALAD score for discriminating any-stage hepatocellular carcinoma in prospective (A, B) and retrospective (C, D) cohorts.

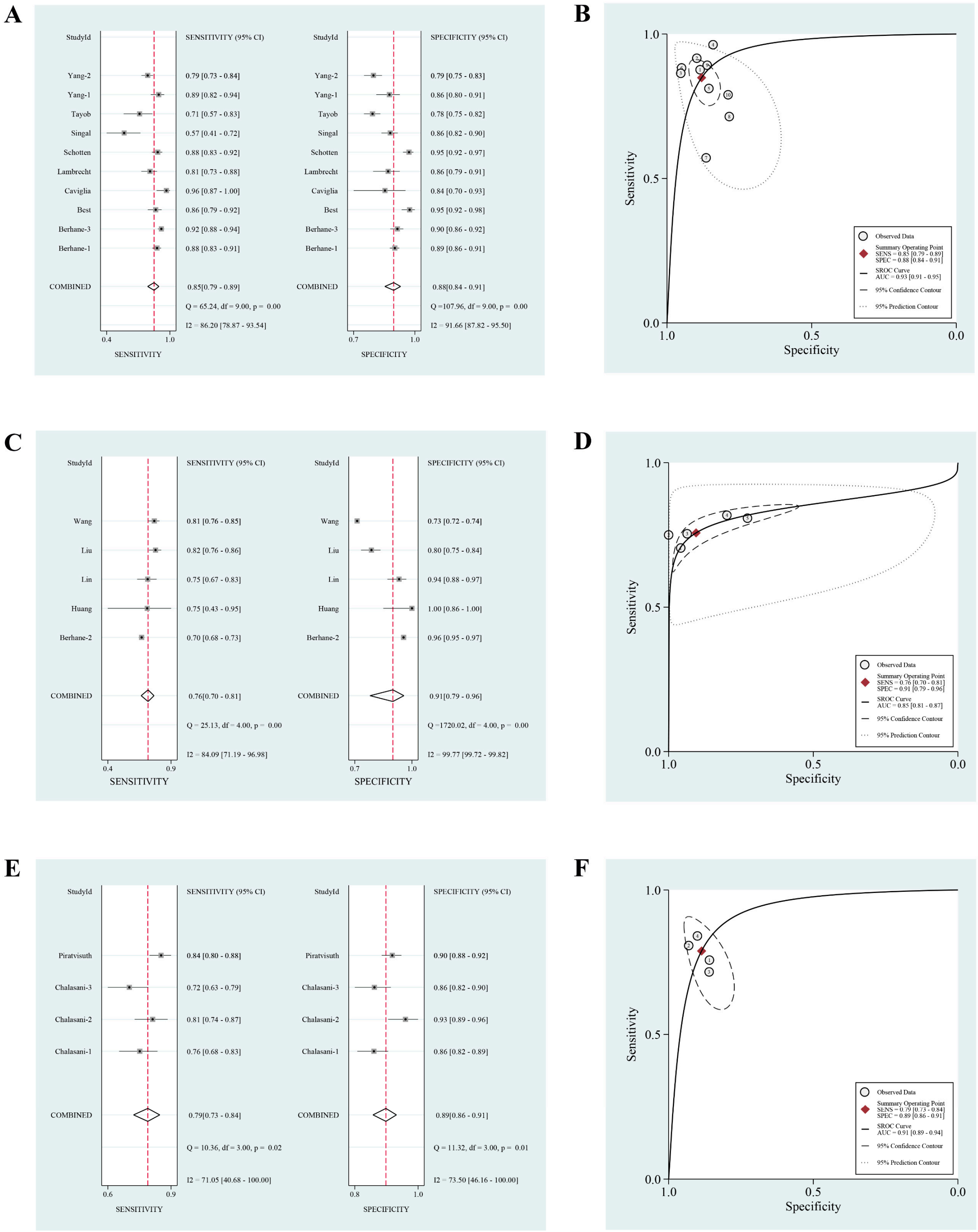

Figure S6. The pooled sensitivity and specificity with Forest plots and summary receiver operating characteristic curve of GALAD score for discriminating any-stage hepatocellular carcinoma in Western countries (A, B), East-Asian countries (C, D), and countries from different continents (E, F).

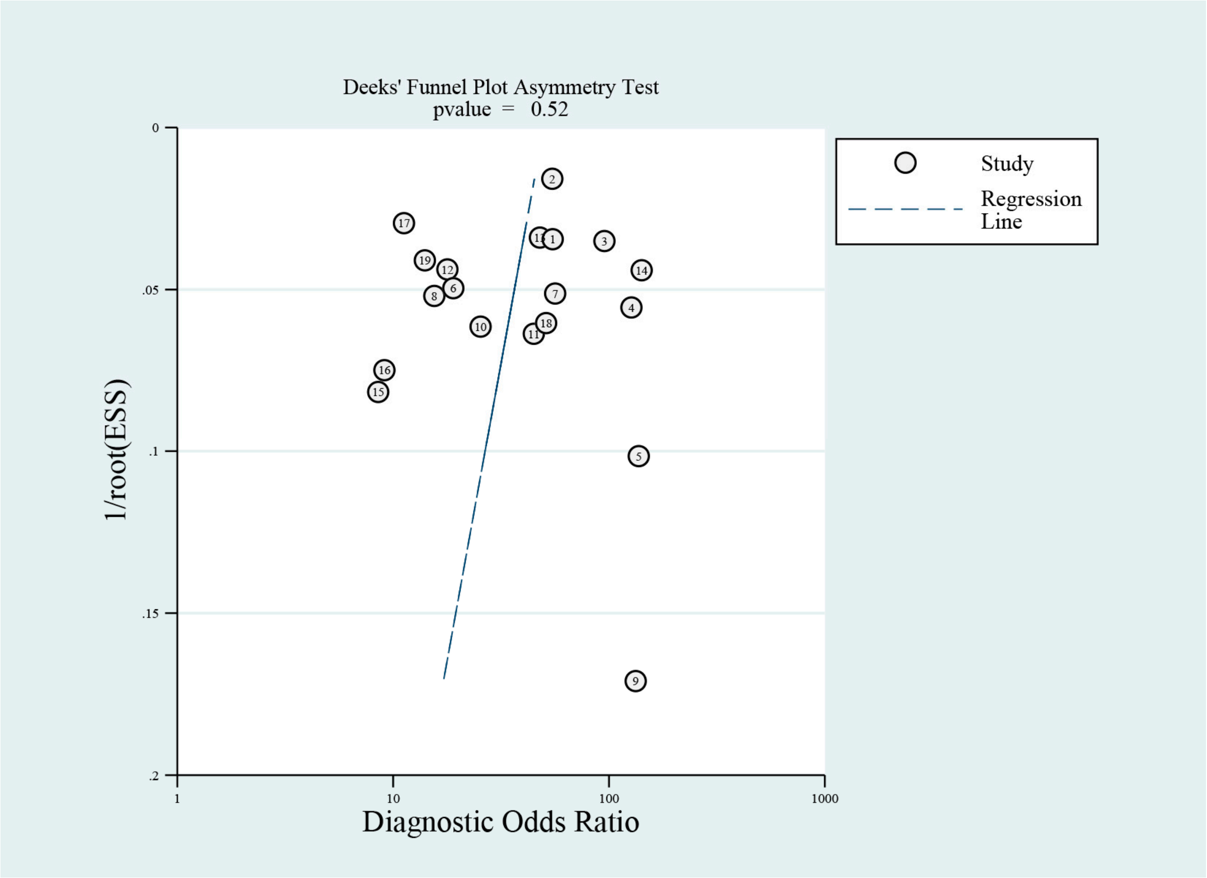

Figure S7. Deeks' plot for publication bias.

Table S1. Search strategies in the *Web of Science*, *PubMed*, *Scopus*, *Ovid*, *Cochrane Library*, and *Embase* databases.

Database	No.	Query	Results
PubMed	#3	#1 AND #2	27
	#2	GALAD	36
	#1	(((Hepatocellular Carcinoma) OR (Liver Cancer)) OR (Hepatoma)) OR (Liver Cell Carcinoma)	340,230
Embase	#3	#1 AND #2	65
	#2	GALAD	80
	#1	('hepatocellular carcinoma') OR hepatoma OR ('liver cancer') OR ('liver cell carcinoma')	268,971
Web of Science	#3	#1 AND #2	53
	#2	TS=(GALAD)	58
	#1	(((TS=(hepatocellular carcinoma)) OR TS=(hepatoma)) OR TS=(liver cancer)) OR TS=(Liver Cell Carcinoma)	438,736
Cochrane Library	#3	#1 AND #2	1
	#2	GALAD	18
	#1	(hepatocellular carcinoma) OR (hepatoma) OR (liver cancer) OR (Liver Cell Carcinoma)	16,941

Scopus	#3	#1 AND #2	223
	#2	GALAD	1,513
	#1	(((Hepatocellular Carcinoma) OR (Liver Cancer)) OR (Hepatoma)) OR (Liver Cell Carcinoma)	1,808,392
Ovid	#3	#1 AND #2	58
	#2	GALAD	88
	#1	(((Hepatocellular Carcinoma) OR (Liver Cancer)) OR (Hepatoma)) OR (Liver Cell Carcinoma)	173,944

Table S2. Proportion of HCC Patients Receiving Different Therapies.

	Berhane-1 [24]	Berhane-2 [24]	Berhane-3 [24]
Transplantation, %	3.6	0	4.8
Resection, %	4.2	32.9	2.6
Ablative, %	3.0	26.9	10.5
TACE, %	26.2	21.0	38.0
Sorafenib/chemotherapy, %	14.3	1.1	16.0
Supportive, %	14.3	13.5	22.7
Other palliative, %	34.5	4.6	5.4

Table S3. Univariable Meta-regression

Parameter	Category	Number cohorts	Sensitivity (95%CI)	*P* value	Specificity (95%CI)	*P* value
studydesign_retro†	Yes	10	0.83 (0.79-0.88)	< 0.01	0.89 (0.86-0.93)	< 0.01
	No	9	0.80 (0.74-0.86)	< 0.01	0.88 (0.83-0.92)	< 0.01
numberszie_500‡	Yes	10	0.82 (0.78-0.87)	< 0.01	0.87 (0.83-0.91)	< 0.01
	No	9	0.81 (0.75-0.87)	< 0.01	0.90 (0.87-0.94)	< 0.01
race_white§	Yes	13	0.83 (0.79-0.87)	< 0.01	0.88 (0.85-0.92)	< 0.01
	No	6	0.79 (0.72-0.86)	< 0.01	0.90 (0.85-0.95)	< 0.01

CI, confidence intervals.

† The study was design retrospectively (Yes) or prospectively (NO).

‡ The number of study cohort was ≥ 500 (Yes) or < 500 (No).

§ The majority of subjects were white (Yes) or not (No).

Table S4. Multivariable Meta-regression

Parameter	Category	LRTChi2	*P* value
studydesign_retro†	Yes	1.04	0.60
	No		
numberszie_500‡	Yes	1.34	0.51
	No		
race_white§	Yes	1.45	0.49
	No		

† The study was design retrospectively (Yes) or prospectively (NO).

‡ The number of study cohort was ≥ 500 (Yes) or < 500 (No).

§ The majority of subjects were white (Yes) or not (No).

Table S5. Alpha-fetoprotein for Detecting Hepatocellular Carcinoma within Barcelona Clinic Liver Cancer 0/A Staging.

Number of study cohorts	Number HCC	Number control	Pooled Sensitivity (95%CI)	Pooled Specificity (95%CI)	Pooled PLR (95%CI)	Pooled NLR (95%CI)	DOR (95%CI)	AUC (95%CI)
5	384	2038	0.38 (0.28-0.49)	0.97 (0.92-0.99)	13.4 (5.3-33.6)	0.64 (0.54-0.75)	21 (8-52)	0.70 (0.66-0.74)

Table S6. GALAD Score for Discriminating Hepatocellular Carcinoma within Barcelona Clinic Liver Cancer 0/A Staging in Cirrhotic Population.

Number of study cohorts	Number HCC	Number control	Pooled Sensitivity (95%CI)	Pooled Specificity (95%CI)	Pooled PLR (95%CI)	Pooled NLR (95%CI)	DOR (95%CI)	AUC (95%CI)
5	365	1113	0.78 (0.66-0.87)	0.80 (0.72-0.87)	4.0 (3.0-5.2)	0.27 (0.18-0.41)	15 (10-20)	0.86 (0.83-0.89)
